# Boric Acid and Borax Protect Human Lymphocytes from Oxidative Stress and Genotoxicity Induced by 3-Monochloropropane-1,2-diol

**DOI:** 10.1007/s12011-024-04060-4

**Published:** 2024-01-13

**Authors:** Hasan Turkez, Ozlem Ozdemir Tozlu, Mehmet Enes Arslan, Cem Baba, Muhammed Melik Saracoglu, Edanur Yıldız, Abdulgani Tatar, Adil Mardinoglu

**Affiliations:** 1 Department of Medical Biology, Faculty of Medicine, Atatürk University, Erzurum, Turkey; 2 Department of Molecular Biology and Genetics, Faculty of Science, Erzurum Technical University, Erzurum, Turkey; 3 Department of Medical Genetics, Faculty of Medicine, Atatürk University, Erzurum, Turkey; 4 Science for Life Laboratory, KTH-Royal Institute of Technology, Stockholm, Sweden; 5 Centre for Host-Microbiome Interactions, Faculty of Dentistry, Oral & Craniofacial Sciences, King’s College London, London, UK

**Keywords:** 3-MCPD, Oxidative status, Boric acid, Borax, Cytotoxicity, Genotoxicity

## Abstract

3-chloro-1,2-propanediol (3-MCPD) is a member of the group of pollutants known as chloropropanols and is considered a genotoxic carcinogen. Due to the occurrence of 3-MCPD, which cannot be avoided in multiplexed food processes, it is necessary to explore novel agents to reduce or prevent the toxicity of 3-MCPD. Many recent studies on boron compounds reveal their superior biological roles such as antioxidant, anticancer, and antigenotoxic properties. In the current investigation, we have evaluated in vitro cytotoxic, oxidative, and genotoxic damage potential of 3-MCPD on human whole blood cultures and the alleviating effect of boric acid (BA) and borax (BX) for 72 h. In our in vitro experiments, we have treated blood cells with BA and BX (2.5, 5, and 10 mg/L) and 3-MCPD (at IC_50_ of 11.12 mg/l) for 72 h to determine the cytotoxic damage potential by using MTT (3-(4,5-dimethylthiazol-2-yl)-2,5-diphenyltetrazolium bromide) and lactate dehydrogenase (LDH) release assays. Oxidative damage was assessed using total antioxidant capacity (TAC) and malondialdehyde (MDA) levels. Genotoxicity evaluations were performed using chromosome aberrations (CAs) and 8-hydroxy deoxyguanosine (8-OHdG) assays. The result of our experiments showed that the 3-MCPD compound induced cytotoxicity, oxidative stress, and genotoxicity in a clear concentration-dependent manner. BA and BX reduced cytotoxicity, oxidative stress, and genotoxicity induced by 3-MCPD. In conclusion, BA and BX are safe and non-genotoxic under the in vitro conditions and can alleviate cytotoxic, oxidative, and genetic damage induced by 3-MCPD in the human blood cells. Our findings suggest that dietary boron supplements may offer a novel strategy for mitigating hematotoxicity induced by xenobiotics, including 3-MCPD.

## Introduction

**Fig. 1:**
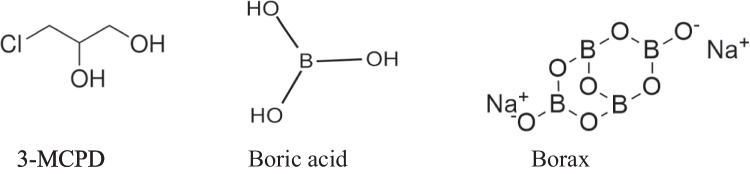
The molecular structures of the tested compounds

3-monochloropropane-1,2-diol (3-MCPD) is a common food borne contaminant and was first identified in acid-hydrolyzed plant proteins. 3-MCPD (Fig. 1) is a well-known human carcinogen detected in a wide variety of foods and ingredients in group 2B, according to the International Agency for Research on Cancer (IARC) [1, 2]. 3-MCPD added to flavor enhancers, cheese, roasting of some grains, especially barley, malt production, during home cooking of prepared foods, cereal products such as bread, soy sauce, meat products, and many food products, including baby foods. Both free and bound forms have been observed in several food products [1, 3, 4]. A tolerable daily intake (TDI) value was determined for 3-MCPD by the European Food Safety Authority (EFSA), corresponding to 2 μg/kg body weight per day. This rate determined by TDI can be exceeded in some cases; for example, infants may consume food containing significant amounts of 3-MCPD [5]. Hence, it is in the focus of food safety authorities due to the possible risks of these substances and minimization strategies are urgently needed to reduce the amount of MCPD [6].

3-MCPD spreads to various organs by crossing the blood-testis and blood-brain barriers. While this causes nephrotoxicity, pulmonary toxicity, hepatotoxicity, and male reproductive toxicity, it may also have negative effects on testicular organogenesis, kidney, immune system, and central nervous system functions [3, 7–9]. Metabolites of 3-MCPD also cause cardiotoxicity by inhibiting glycolysis and alter circadian clock mechanisms [10, 11]. Recent proteomic and transcriptomic studies indicated that glutathione metabolism affected by 3-MCPD and oxidative stress occurred after 3-MCPD exposure in several organs in rats [12]. Besides, 3-MCPD-induced genotoxic damage after treatment for 24 h on rat kidney NRK-52E proximal tubular epithelial and human HEK-293 embryonic kidney cells was determined by alkaline comet assay [13]. On the contrary, 3-MCPD did not exerted genotoxic damage potential as monitored using in vivo bone marrow micronucleus and unscheduled DNA synthesis as genotoxicity end-points on rats [14]. Hence, the underlying in vitro and in vivo genotoxicity mechanisms by 3-MCPD exposure are still unclear and need further investigations.

Boron (B) is not considered an essential trace element for bacteria, fungi, plants, as well as algae but not for humans yet. But recent studies support its essentiality on animals and humans [15]. B compounds have a wide range of applications, including fertilizers, insecticides, cosmetics, pharmaceuticals, food supplements, cleaning products, and personal care items [16–18]. For an extended period, B-containing compounds were neglected in clinical research due to the prevailing belief in their toxicity, primarily linked to their use in ant poisoning. Presently, this perception has been debunked, and boron-containing compounds are generally recognized as non-toxic [19]. B-containing compounds especially boric acid (BA, Fig. 1) and borax (BX, Fig. 1) exhibited beneficial actions on human health. Up to now, the reported key biological benefits by BA and BX included anti-microbial[20], anti-oxidative [21], anti-inflammatory [22] anti-mutagenic [16, 23], anti-cancer [24, 25], neuroprotective [26], hepato-protective [27, 28], reno-protective [29], metal chelating [30], and wound healing [31] activities. Based on these well-established biological effects by B-containing compounds, boron-based hybrids are considered novel structural scaffolds for the development of innovative drugs for the management of acute, chronic, and rare diseases as well as cancers.

In vivo proteomic and transcriptomic data indicated that exposure to 3-MCPD triggered oxidative stress and affected glutathione (GSH) metabolism in rats [12]. Recent literature executed that BA and BX displayed geno-protective action against several chemical agents such as aflatoxin B1, trichloroacetic acid, and cyclophosphamide via strengthening the antioxidant capacity of liver and blood tissues [32–35]. Indeed, in vitro application of BA and BX (< 80 mg/L) led to elevation of total glutathione (T-GSH) and total antioxidant capacity of cultured human whole blood cells [21]. Similar to this previous in vitro finding, supplementation with B (as BA) yielded higher levels of GSH in blood tissue of rats [36]. Hence, BA and BX may enhance the antioxidant defense mechanism and ameliorate the cytotoxic, oxidative, and genotoxic damage by 3-MCPD. The available literature data have shown that no study has been carried out on the protective effects of BA and BX against 3-MCPD-induced toxicity in cultured human blood cells. Therefore, primary human blood cells were used as the cell model in our experiment to investigate the cytotoxicity, genotoxicity, and oxidative damage of 3-MCPD and the protective effect of two common B-containing compounds, boric acid and borax.

## Materials and Methods

### Experimental Design

Whole blood samples were collected from five healthy, non-smoking male volunteers within the age group of 26–32 years in heparinized vacutainers (Greiner Bio-One, Austria). Within 2 hours of sample collection, whole blood cultures were established. Human peripheral blood cultures were established using a slightly modified procedure as previously described [37]. In brief, the 0.6 mL of heparinized blood was cultured in 6.5 mL of culture medium (chromosome medium B, Biochrom, Leonorenstr. 2-6.D-12247, Berlin) with phytohemagglutinin (5 μg/mL, Biochrom). 3-MCPD (0–640 mg/L, CAS No.: 96-24-2, Merck) was dissolved in dimethyl sulfoxide (DMSO, 0.5%) and different concentrations were applied to the culture tubes to determine its IC_50_ value. DMSO was < 1% and did not alter the viability of cells. The cell cultures were then treated with various concentrations (2.5, 5, and 10 mg/L) of boric acid (CAS No.: 10043-35-3) and borax (CAS No.: 1303-96-4) were used against 3-MCPD. Boric acid and borax were provided from Eti Mine Works (Ankara, Turkey) and their concentrations were selected to previous reports [26, 30]. Triton-X-100 (%1), ascorbic acid (10^–5^ M), hydrogen peroxide (2.5 × 10^–5^ M), and mitomycin C (10^–7^ M) were also used as positive controls for MTT/LDH, TAC, MDA, and CA/8-OHdG assays, respectively [38]. All in vitro experiments were conducted due to rules of the World Medical Association

### Cytotoxicity Testing

To determine cell viability, after treatment with boron compounds and 3-MCPD for 72 h, commercially available 3-(4,5-dimethylthiazol-2-yl)-2,5 diphenyltetrazolium bromide (MTT) kit (MTT Cell Proliferation Kit, Cayman, Ann Arbor, MI, USA) was applied to cell cultures following the manufacturer’s instructions. In summary, MTT solution was added to cell cultures, incubated at 37 °C for 3 h, then dimethyl sulfoxide (DMSO) (Sigma-Aldrich) was used to dissolve formazan crystals. A plate reader was assisted to analyze the cultures and read at 570 nm [39]. IC_50_ value of 3-MCPD was calculated using probit analysis based on results of MTT assay.

The LDH test kit available from Cayman Chemical Company (MI, USA) was applied following the manufacturer’s instructions. Different concentrations of boron compounds and 3-MCPD were applied to the wells for 72 h after laundering the cells in 96-well plates. The 96-well plate was then centrifuged at 400 g for 5 min to eliminate the compounds in the wells. After centrifugation was finished, 100 μL of supernatant and 100 μL of the reaction mixture were added to another well plate and then incubated for 30 min at room temperature. Absorbance was read at 490 nm with the aid of a microplate reader [40].

### Oxidative Analysis

For determining the total antioxidant capacity, commercially available TAC (Rel Assay Diagnostics, Gaziantep, Turkey) kits were used. Measurements from cellular samples were carried out due to the manufacturer’s recommended procedure [41]. Besides, the level of MDA was determined in plasma samples by the thiobarbutiric acid (TBA) method which modified from previously reported methods [42, 43]. Peroxidation was determined via measuring the production of a pink chromogen compound which reflects the MDA in combination with TBA at 532 nm [44].

### Genotoxicity Testing

Human blood cultures were exposed to 3-MCPD, boron compounds, and their combinations and cultured for 72 h. At the 70th hour of harvest, 0.1 mL of colchicine (0.2 mg/mL, Sigma) was added to the culture flask. Centrifugation was performed, cells were collected and cultures were treated with hypotonic KCl (0.075 M KCl, 37.4 °C) solution. Cells were centrifuged again and treated with 3:1 methanol:acetic acid solution and this process was repeated three times. The resulting cells were suspended and dripped onto clean slides. To prepare the slides, three to five drops of fixed cell suspension were placed on an ice-cold, wet slide and allowed to air dry. Slides were stained with Giemsa stain in phosphate buffer pH 6.8. For treatments, 30 metaphase analyzes were performed to detect chromosomal abnormalities [45]. The recommendation of Environmental Health Criteria 46 for environmental monitoring of human populations was followed to classify differences on chromosomes, such as chromosome breakage and chromosomal spacing [46].

The amount of 8-OHdG adducts was determined to measure DNA oxidation. DNA was digested after incubation with DNAase I, endonuclease, and alkaline phosphatase enzymes. The amount of 8-OHdG was measured using high-performance liquid chromatography (HPLC) with electrochemical detection [47, 48].

### Statistical Analyses

Statistical analysis was carried out using SPSS statistics 25.0 software (Statistical Package for the Social Sciences Inc, Chicago, USA). All tests were performed in five different repetitions. The obtained data was analyzed using ANOVA test followed by Duncan’s test. Probit regression analyses was also performed to determine the IC_50_ concentration of 3-MCPD using SPSS. And, the level of *p* < 0.05 was accepted as significantly different.

## Results

**Fig. 2:**
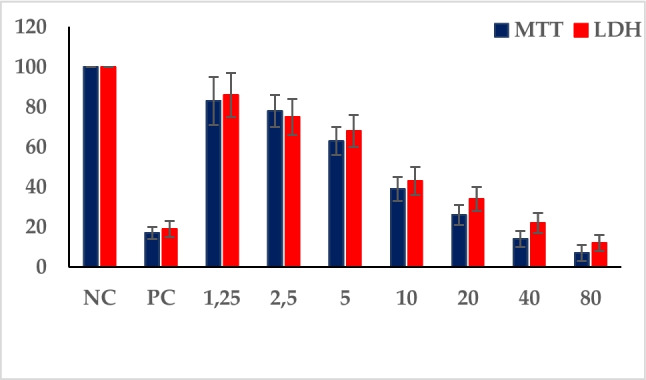
The cell viability rates after exposure to 3-MCPD in human whole blood cultures for 72 h using MTT and LDH assays

In this study, MTT as a colorimetric method and LDH as an enzymatic method were used to assess the cell viability after treatment with BA, BX, and 3-MCPD in human whole blood cultures. One-percent solution of Triton-X was used as a positive control, and reduced cell viability percentages to 17.44% and 29.15% in the MTT and LDH assays, respectively. The calculated 3-MCPD IC_50_ value (using MTT results) for human blood cells was 11.12 mg/L (Fig. 2).

**Fig. 3:**
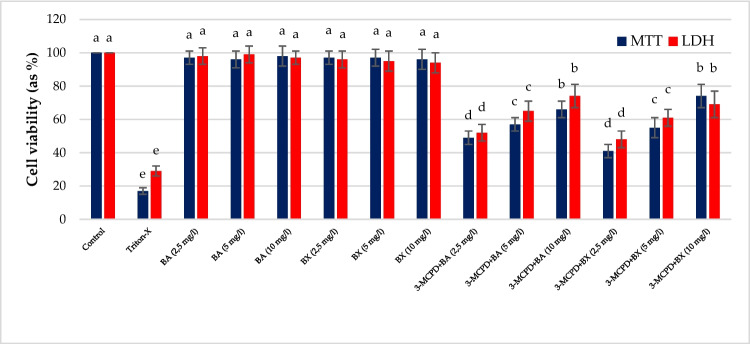
Cell viability rates in cultured human peripheral blood cells after treatment with different concentrations of two boron compounds (BA and BX) plus 3-MCPD. Different letters on the columns present statistical difference among each other at a level of *p* < 0.05

MTT and LDH results for BA and BX revealed no statistically significant difference from the negative control. Our results indicate that BA and BX, including at the highest concentrations (10 mg/L), did not exert cytotoxic effects on cultured human blood cells. Contrary to these values, MTT and LDH results of cells treated with 3-MCPD showed that this substance resulted in significant cell viability reductions compared to negative control (Fig. 3). When we look at the results of BA treatment applied to 3-MCPD-treated blood cultures, it was observed that BA increased cell viability more than doubled as compared to the untreated culture (*p* < 0.05). As the concentration increases, the percentage of cell viability increases and the concentration with the highest cell viability is expressed as 10 mg/L. The application of BX with 3-MCPD also increased cell viability as compared to the untreated culture (*p* < 0.05). When MTT and LDH results for BA and BX are compared, BA is found to be more effective at 2.5 mg/L applied concentration. Moreover, BX gives higher cell viability at 5 mg/L applied concentration (Fig. 3).

**Fig. 4:**
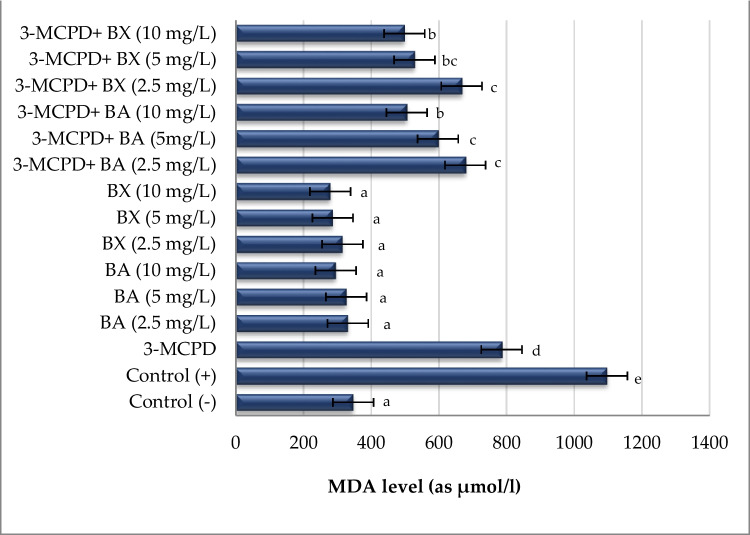
The effect of 3-MCPD and boron treatments on MDA levels in cultured human blood cells. Different letters on the columns present statistical difference among each other at a level of *p* < 0.05

**Fig. 5:**
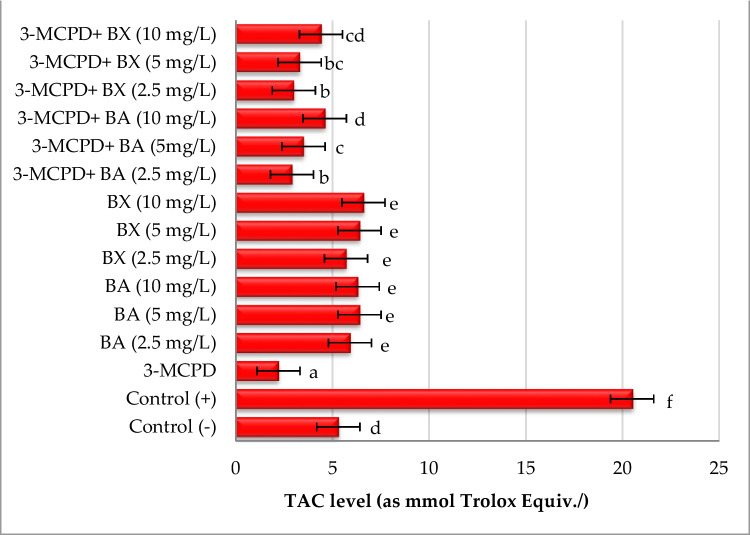
The effect of 3-MCPD and boron treatments on TAC levels in cultured human blood cells. Different letters on the columns present statistical difference among each other at a level of *p* < 0.05

Two biochemical analyses, MDA and TAC, were used to determine and evaluate the oxidative effect of BA and BX on culture in human whole blood cultures exposed to 3-MCPD. Ascorbic acid was used as a positive control for TAC assay and H_2_O_2_ was used as a positive control for MDA assay. It was observed that 3-MCPD caused a significant decrease (approximately 59%) in TAC level in comparison to untreated cell culture, which indicated a remarkable oxidative stress generation. Likewise, a significant increase (*p* < 0.05) of MDA level (approximately 126%) was determined in 3-MCPD-treated culture. On the contrary, alone treatment with BA and BX led to increases of TAC levels without elevating MDA levels. Moreover, when the efficiencies of two boron compounds were evaluated, it was observed that there were significant decreases in MDA levels after treatment with BA and BX as compared to the positive control. Similarly, 10 mg/L of BA and BX treatment supported TAC levels in rates of 109% and 100%, respectively. The antioxidative action by BA and BX against 3-MCPD-induced suppression of TAC levels was clearly in concentration-based manner (Figs. 4 and 5).

**Fig. 6:**
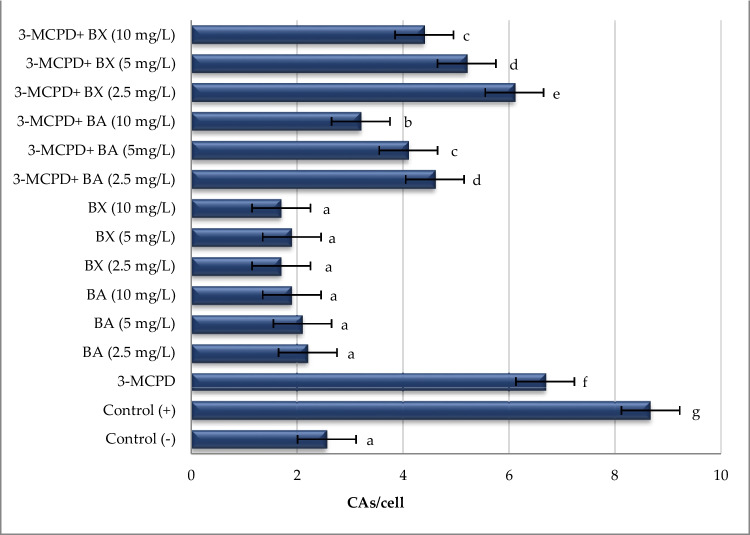
The rates of CAs in cultured human peripheral blood cultures after treatment with BA and BX plus 3-MCPD for 72 h

**Fig. 7:**
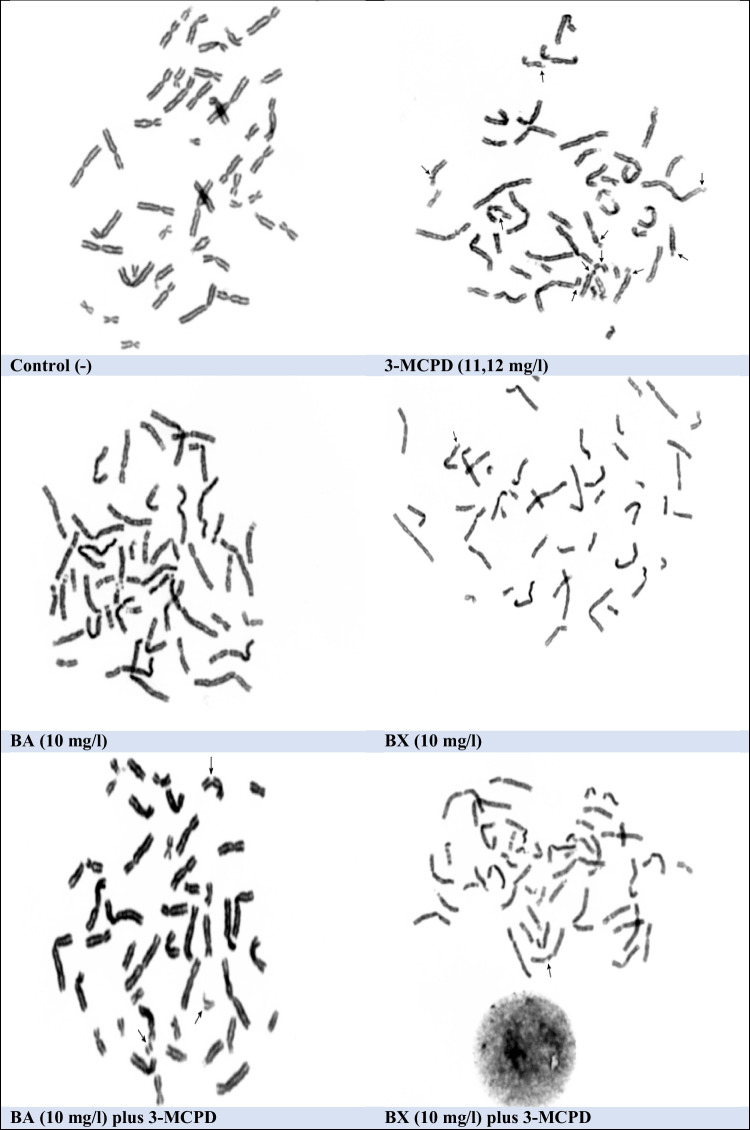
The sample metaphases from the human blood cultures after treatment with BA and BX plus 3-MCPD for 72 h. Arrows show chromosomal aberrations (breaks and gaps, × 1000)

**Fig. 8:**
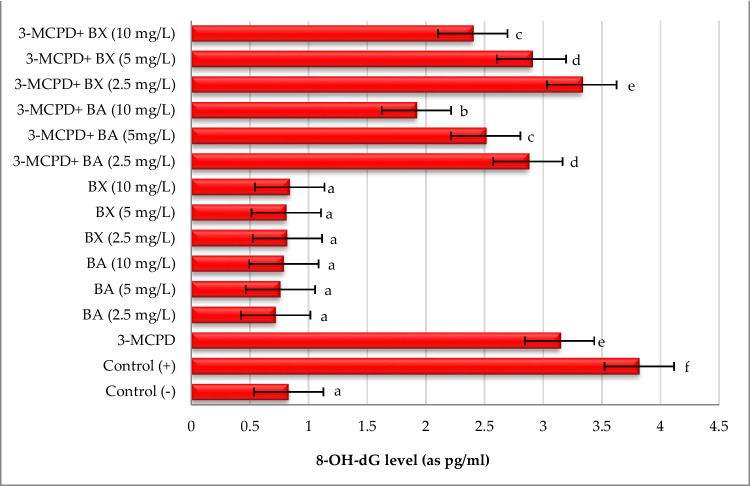
The amounts of 8-OHdG in cultured human peripheral blood cultures after treatment with BA and BX plus 3-MCPD for 72 h

The genotoxicity potentials of two boron compounds and 3-MCPD borinium compounds were evaluated in cultured human lymphocytes by CA and 8-OHdG assays and the obtained results were presented in Figs. 6, 7, and 8. It was found that in vitro exposure to BA and BX did not cause significant (*p* > 0.05) increases in CA regardless of concentrations, indicating that tested boron compounds (BA and BX) have a non-genotoxic nature. On the contrary, both MMC (positive control) and 3-MCPD increased the CA frequency in approximately 3.4- and 2.6-fold. However, it was determined that treatment with boron compounds against 3-MCPD toxicity positively improved the increases of CA level caused by 3-MCPD. In fact, it was found that the CA frequency decreased in parallel with the increase in the concentration of BA and BX (Figs. 6 and 7). Likewise, treatment with MMC alone and 3-MCPD alone led to increases of 8-OHdG levels in approximately 4.6- and 3.8-fold. On the contrary all cultures treated with BA and BX did not alter the 8-OHdG levels in comparison to untreated cultures. Moreover, remarkable reductions in 8-OHdG levels were observed in groups treated with alone BA and BX treatments as compared BA/BX plus 3-MCPD treated group, especially with the highest concentrations (10 mg/L) of BA or BX. When boric acid and borax were compared among themselves, it was observed that BA exhibited a higher ameliorative effect than BX. 8-OHdG levels also supported the results of CA as seen in Fig. 8.

## Discussion

Vegetable oils are processed in various ways in the several industrial domains to remove components that will adversely affect important parameters such as flavor, appearance, and shelf stability [49]. After the deodorization step in the refining process the formation of fatty acid esters of 3-MCPD, 2-chloro-1,3-propanediol (2-MCPD), and glycidol are occurred [50]. Hence, 3-MCPD is considered a main source of contamination during the food and ingredient processing and possess health risks on animals and humans [51]. In the content of this investigation, the obtained results by cytotoxicity testing indicated that 3-MCPD-induced cell death was associated with mitochondrial injury and disturbance of cellular metabolic events (MTT assay) as well as necrosis due to cell membrane damage (LDH release assay). In accordance to our finding, a recent study revealed that 3-MCPD led to activation of cell death signaling via impairment of mitochondrial oxidative phosphorylation system in cultured human embryonic kidney (HEK) 293 cells and male C57 mice [51, 52]. Again, the induction of necroptosis was suggested as associated with cytotoxicity by 3-MCPD on the rat renal proximal tubular NRK-52E cells [53]. In similar to human blood cultures used in this study, different concentrations of (0–5 mM) 3-MCPD also reduced the cell viability rates and induced intracellular LDH leakage as compared to untreated HEK293 cells [54].

Previous reports indicated that oxidative stress might commit a major role in toxicity by 3-MCPD. Nominately, 3-MCPD caused to damage of renal antioxidant capacity in experimental rats. The in vivo oxidative damage by 3-MCPD was occurred via elevating glutathione and MDA levels as well as decreasing TAC levels in rat kidney tissues [55]. In another in vivo study, it was reported that low doses (1 and 10 mg/kg b.w.) of 3-MCPD generated oxidative stress in brain, kidney, and testes tissues of mice for 28 days by inclining irreversible oxidation of the redox sensor protein named as DJ-1 [12]. Due to its antioxidative feature, DJ-1 is known to play key roles in multiplexed signaling pathways including the activation of extracellular signal-regulated kinase (ERK1/2) pathway and inhibition of apoptosis signal-regulating kinase 1 (ASK1) [56]. In addition to these in vivo studies, several in vitro studies propounded that 3-MCPD generated intracellular reactive oxygen species (ROS) in cultured HEK293 and HK-2 cells [57, 58]. And, elevated intracellular Fe^2+^ levels and lipid peroxidation were reported after exposure of 3-MCPD to human umbilical vein endothelial cells (HUVEC) [59]. And, in vivo exposure to 3-MCPD led to inhibition of NF-E2-related factor 2 (Nrf2) expression, disrupted Ca^2+^ homeostasis, and triggered the oxidative stress [60, 61]. In line with the previous literature data on oxidative damage potential by 3-MCPD, our findings firstly revealed that this contaminant induced oxidative damage on human blood cells via suppressing TAC levels and generating lipid peroxidation (elevated MDA levels).

Our findings asserted that 3-MCPD alone led to increases of CA rates and 8-OHdG levels in cultured human blood cells. In accordance with our finding, 3-MCPD was identified as genotoxic or mutagenic in various in vitro genotoxicity testing studies involving Ames Salmonella/microsome mutagenicity, sister chromatid exchange (SCE), and mouse lymphoma assays [3, 62]. The genotoxic damage potential of 3-MCPD was also confirmed using alkaline comet assay on NRK-52E and HEK-293 cells and observed increased DNA damage after exposure of 2 mg/mL of 3-MCPD when compared to control values [17]. To the contrary, increasing pieces of evidence executed that 3-MCPD exerted non-genotoxic under in vivo conditions [63, 64]. However, there is so limited information available to explain the controversial findings of in vitro and in vivo genotoxicity by 3-MCPD. This *in vitro* positive and *in vivo* negative situation due to 3-MCPD exposure could be explained via substrate channeling effect which implicate detoxification of in vitro genotoxic compounds in the presence of in vivo detoxifying actions [65]. Our findings exerted that 3-MCPD-induced oxidative stress (TAC suppression, MDA elevation) might be a main contributing factor for the increased CA rates and 8-OHdG levels determined in the present investigation. Numerous studies have established a robust association between the occurrence of lipid peroxidation and genetic damage, as evidenced by increased frequencies of chromosomal aberrations (CAs) and elevated levels of 8-OHdG. [66–71].

Previous toxicity studies clearly revealed the harmful effects of 3-MCPD and exposure to 3-MCPD seems to be inevitable. Because vegetable oils are the main source of fat in many foods currently in use, the presence of this contaminant has been recognized as a potential health risk [50]. In parallel with the fact that 3-MCPD exposure is inevitable, there is an urgent need to reduce, eliminate, or prevent the harmful effects of this substance. In this regard, our investigation firstly reveals that treatment with boron compounds like BA and BX ameliorate cytotoxicity induced by 3-MCPD and prevents genotoxic damage via decreasing lipid peroxidation and supporting antioxidant capacity in human blood cells. Our results provided considerable data regarding the protective roles by BA and BX, and the conceivable underlying mechanism of their protective action. In this respect, a previous study indicated that boron-containing compounds such as BA and BX prominently supported the antioxidant capacity of human blood cell until the applied concentrations of 20 mg/L [21]. Correlatively, boron-containing compounds exhibited anti-genotoxic action due to their tissue antioxidant defenses strengthening potential via (I) leading increases of antioxidant enzymes activities like glutathione peroxidase, superoxide dismutase, and catalase [30]; (II) elevating glutathione production [22]; (III) activating Nrf2 and the antioxidant response elements which regulate the redox homeostasis during oxidative stress [72, 73]; and (IV) reducing the amounts of intracellular ROS and levels of Ca^+2^ ions [74]. These suggested different antioxidative action manners were cleared that antioxidant roles of boron compounds could be suggested as main plausible mechanism for protective effects of BA and BX against in vitro cytotoxic, oxidative, and genotoxic damages induced by 3-MCPD on human blood cells.

In conclusion, our results showed that treatment with BA and BX not only decreased the levels of oxidative stress endpoints like MDA and suppression of TAC but also significantly minimized the levels of genotoxicity endpoints like CA and 8-OHdG. Upon examination of the two compounds, it was noted that BA exhibited greater ameliorative effect than BX against in vitro cytotoxic, oxidative, and genotoxic damages induced by 3-MCPD on human blood cells. These findings are outstanding because boron compounds, especially BA and BX, may be used as safe and natural dietary supplements for alleviating cytotoxic and genotoxic effects by multiplexed mutagenic and carcinogenic substances.

## Data Availability

No datasets were generated or analysed during the current study.
